# Nanofiltration and Tight Ultrafiltration Membranes for Natural Organic Matter Removal—Contribution of Fouling and Concentration Polarization to Filtration Resistance

**DOI:** 10.3390/membranes7030034

**Published:** 2017-07-02

**Authors:** Joerg Winter, Benoit Barbeau, Pierre Bérubé

**Affiliations:** 1 Department of Civil Engineering, The University of British Columbia, 6250 Applied Science Lane, Vancouver, BC V6T1Z4, Canada; jwinter@civil.ubc.ca; 2 Department of Civil, Geological and Mining Engineering, École Polytechnique de Montréal, Montréal, QC H3T 1J4, Canada; benoit.barbeau@polymtl.ca

**Keywords:** nanofiltration, tight ultrafiltration, concentration polarization, fouling, natural organic matter

## Abstract

Nanofiltration (NF) and tight ultrafiltration (tight UF) membranes are a viable treatment option for high quality drinking water production from sources with high concentrations of contaminants. To date, there is limited knowledge regarding the contribution of concentration polarization (CP) and fouling to the increase in resistance during filtration of natural organic matter (NOM) with NF and tight UF. Filtration tests were conducted with NF and tight UF membranes with molecular weight cut offs (MWCOs) of 300, 2000 and 8000 Da, and model raw waters containing different constituents of NOM. When filtering model raw waters containing high concentrations of polysaccharides (i.e., higher molecular weight NOM), the increase in resistance was dominated by fouling. When filtering model raw waters containing humic substances (i.e., lower molecular weight NOM), the increase in filtration resistance was dominated by CP. The results indicate that low MWCO membranes are better suited for NOM removal, because most of the NOM in surface waters consist mainly of humic substances, which were only effectively rejected by the lower MWCO membranes. However, when humic substances are effectively rejected, CP can become extensive, leading to a significant increase in filtration resistance by the formation of a cake/gel layer at the membrane surface. For this reason, cross-flow operation, which reduces CP, is recommended.

## 1. Introduction

Over the past 20 years, membrane filtration has been increasingly implemented in water purification processes [1]. This increase has mainly been driven by the decreasing costs of membrane systems, and increasingly stringent drinking water quality regulations. In drinking water treatment applications, ultrafiltration membranes (UF) can effectively remove particulate contaminants, such as protozoa and bacteria, from raw water sources. However, effective removal of natural organic matter (NOM) and viruses is generally limited [2,3]. This is because the molecular weight cut off (MWCO) of UF membranes typically used in drinking water treatment applications is relatively large (i.e., greater than 100,000 Da). Membranes with a MWCO of less than 10,000 Da are required to effectively remove NOM and all pathogens [4,5,6,7,8]. The present study investigated the use of membranes with MWCOs ranging from 300 to 8000 Da for the removal of NOM. In this range, membranes are commonly referred to as nanofiltration (NF) membranes (i.e., with a typical MWCO range of 200–1000 Da) and tight UF membranes (i.e., with a typical MWCO range of 1000 to 10,000 Da). The removal of NOM from source waters is of importance in drinking water treatment because it can cause color, taste, and odor issues, increase chlorine demand, as well as contribute to disinfection by-product formation and microbial regrowth in distribution systems [9,10,11,12,13,14]. Because NF and tight UF membranes can achieve sufficient primary disinfection (i.e., >4-log removal of all pathogens) and extensive removal of NOM, they can provide effective and comprehensive drinking water treatment in a single step. A simple, single-step approach is of particular interest for small/remote communities, because of the limited financial and technical resources generally available to implement and operate water treatment systems.

The use of NF and tight UF membranes in drinking water treatment applications is still limited. This is mainly because spiral wound configurations, which generally require extensive pre-treatment and a high trans-membrane pressure, have historically been used for NF and tight UF membrane applications. In addition, fouling control measures, such as backwashing or surface scouring, cannot be applied to spiral wound configurations. Recent developments in NF and tight UF membrane configurations, such as hollow fiber configurations, promise to address some of these limitations [15,16,17,18]. Frank et al. have also reported that a substantially higher permeability can be maintained using hollow fiber configurations [16]. However, other studies have reported that hollow fiber configurations are less effective in mitigating concentration polarization (CP) than spiral wound configurations [19,20]. Because CP and fouling are still major challenges in the application of NF and tight UF membranes for drinking water treatment, insight into CP and fouling is necessary to develop recommendations for the design and operation of NF and tight UF membranes, and to enable greater adoption of this advanced treatment technology.

Fouling occurs as material that is retained at the membrane surface or inside the membrane pores, accumulates, and increases resistance to the permeate flow. Material accumulates when the permeation drag—the force that transports potential foulants towards the membrane—is greater than forces acting in the opposite direction (i.e., away from the membrane) [21,22]. NOM is generally considered to be a main contributor to membrane fouling in drinking water treatment applications [4,23,24,25,26,27]. The extent of fouling is not necessarily proportional to the total amount of NOM retained, but is rather governed by the retention of specific NOM fractions [28,29,30,31]. The NOM fractions that have been reported to be mainly relevant to membrane fouling are biopolymers and humic substances, which are mainly of aquatic and terrestrial origin, respectively [32,33,34]. For UF membranes, the biopolymer fraction of NOM generally contributes the most to fouling [35,36,37,38]. Fouling due to humic substances is generally not as extensive, although it is more difficult to control hydraulically (e.g., by backwashing) [25,35]. The extent and the hydraulic reversibility of UF fouling can also be affected by other constituents in raw waters, notably calcium [25,35,39]. Calcium can form bridges between NOM molecules, between the membrane surface and NOM, as well as contribute to aggregation of NOM by charge destabilization [25,35,39]. Biopolymers have also been reported to substantially contribute to fouling of NF and tight UF membranes [28,38,40]. Humic substances, although effectively rejected, have not been reported to substantially contribute to fouling of NF membranes [41]. The limited contribution of humic substances to fouling has been attributed to charge repulsion effects, which enhance the back transport of humic substances in cross-flow systems [41,42]. The extent to which electrostatic repulsion contributes to fouling control has been demonstrated to increase with the ratio of cross-flow velocity to permeate flux [39]. Therefore, the effect of electrostatic repulsion on fouling due to humic substances, is likely to be substantially less pronounced in dead-end systems. The reported effects of calcium on fouling in NF and tight UF membranes systems have been inconsistent, and likely depend on the concentration in the solution being filtered [29,39,43,44].

CP, resulting from the accumulation of dissolved material rejected by the membrane, can also increase the resistance to permeate flow [45,46,47]. Unlike the situation with fouling, material does not deposit on the membrane surface or inside the membrane pores, but accumulates in proximity to the membrane surface. CP is characterized by an equilibrium between the convective transport of material towards the membrane, and the diffusive transport of retained material away from the membrane; a steady state that is expected to develop within the first minutes of filtration [29,45,48]. CP can affect the resistance to permeate flow by increasing the viscosity, the osmotic pressure of the solution being filtered, and the back-diffusion of retained solutes [45]. If CP effects become extensive, solutes at the membrane surface can reach a critical concentration (i.e., solubility limit), beyond which they form a cake/gel layer which fouls the membrane surface [26,46,47,48]. In literature, this critical concentration is also referred to as the “gel concentration” [46,47]. If a material reaches the critical concentration, the back-diffusion of material is limited by the formation of a cake/gel layer. Under such conditions, the convective transport towards the membrane becomes greater than the diffusive transport away from the membrane, and fouling occurs, which increases the resistance to permeate flow throughout the filtration phase. According to the Stokes–Einstein equation, the diffusivity and the diffusive transport of material depends on its size. NOM ranges in size from a few hundred Daltons (low molecular weight acids and neutrals) to over 20,000 Da (biopolymers) [39,49,50,51]. The size of NOM also generally increases with ionic strength, and decreases with pH of a solution, due to changes in the shape of NOM from uncoiled to coiled [52]. CP due to the retention of salts in Reverse Osmosis (RO) and NF systems has been extensively investigated [19,53,54,55,56]. However, only a few studies have investigated CP due to the retention of NOM in NF membranes. These studies have indicated that NOM can result in CP, and therefore, affect system performance [26,57]. Also, comprehensive knowledge on the effect of (i) the type of NOM (e.g., polysaccharides and humic substances), and (ii) the membrane MWCO on the contribution of CP to the total increase in filtration resistance, is not currently available. This is a knowledge gap that limits the ability to develop recommendations for the optimal design and operation of NF and tight membrane systems for drinking water treatment.

The objective of the present study was to quantify the contribution of fouling and CP to the total increase in resistance during filtration of model raw waters containing polysaccharides, humic substances, and a mixture of both (as typical constituents of NOM in natural surface waters), and to identify conditions under which an extensive increase in filtration resistance occurs. Since fouling and CP are not only expected to depend on the raw water characteristics, but also on the membrane’s selectivity, the impact of the MWCO of a membrane on CP and fouling was also investigated. As previously discussed, CP is expected to develop rapidly within the first minutes of filtration [39,45]. However, this has never been investigated for NF and tight UF membranes in drinking water applications.

## 2. Material and Methods

### 2.1. Experimental Approach

Model raw waters of various compositions were considered. The biopolymer and humic material content of the raw waters were modeled using polysaccharide alginate derived from brown algae (Sodium Alginate, Sigma Aldrich, Oakville, ON , Canada) and Suwannee River NOM (SRNOM) 2R101N (International Humic Substances Society, St. Paul, MN, USA), respectively. Model raw waters with three different NOM compositions were considered: (i) alginate, (ii) SRNOM, and (iii) mixtures of SRNOM and alginate with a carbon mass ratio of 4:1. The ratio of 4:1 in the mixtures of SRNOM and alginate was selected based on humic substances to biopolymers ratios obtained from size exclusion chromatography (SEC) analyses of local surface water (Jericho Pond, Vancouver, BC, Canada) and is also consistent with the ratios reported in literature, which indicates that humic substances account for 70–80% of the NOM in natural waters [32]. All three model raw water compositions were tested at two dissolved organic carbon (DOC). concentrations: 5 mg/L, and 10 mg/L.

To prepare the model raw waters, NOM surrogates (i.e., the polysaccharide alginate and/or SRNOM) were dissolved in Milli-Q laboratory water (MilliporeSigma, Temecula, CA, USA). Sodium bicarbonate at a concentration of 1 mM was added as a buffer. This concentration of bicarbonate also corresponds to an alkalinity that is within a range typical of that present in natural source waters [58]. The retention of alkalinity during filtration ranged from approximately 2% to 40% (depending on the membrane MWCO), and the impact of alkalinity rejection on CP and resistance during filtration was calculated [28], and found to be negligible relative to the overall increases in resistance observed in the present study (results not shown), and hence, is not further discussed. The pH was then adjusted to 7.0 (± 0.1), using sodium hydroxide or hydrochloric acid, as needed. Prior to all filtration tests, all model raw water solutions were filtered through a 0.45 μm nitrocellulose filter. Milli-Q laboratory water was used for clean water flux tests prior- and post-filtration of model raw waters. The pH of the Milli-Q laboratory water used for clean water flux tests was also adjusted to 7.0 (±0.1), using sodium hydroxide.

**Figure 1:**
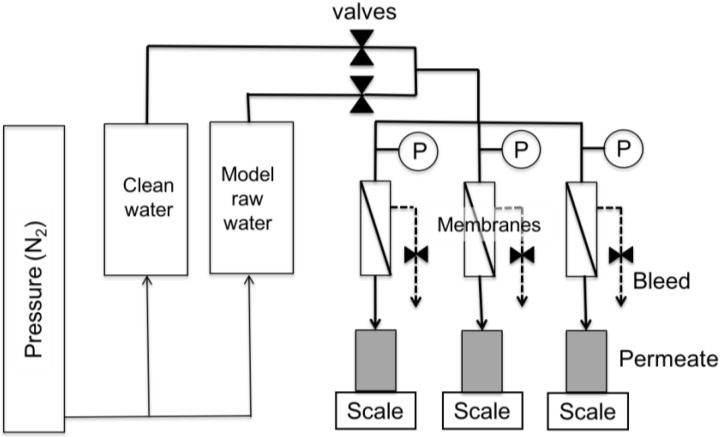
Experimental setup.

The experimental setup, illustrated in Figure 1, consisted of feed vessels, membrane cells, bleed lines and permeate collection systems. Two feed vessels were used, one containing clean water and the other containing a model raw water. The pressure applied to both vessels was equal, enabling the feed to the membrane to be switched between model raw water and clean water without causing any pressure variation in the membrane cells. Three CF042 membrane cells (Sterlitech, Seattle, WA, USA) were used in parallel. These accommodated flat sheet membrane coupons, 39.2 mm wide and 85.5 mm long. Flat sheet, thin film composite polyamide (PA) membranes with nominal MWCOs of approximately 300 Da (DK series, GE Osmonics, Trevose, PA, USA), 2000 Da (GH series, GE Osmonics) and 8000 Da (GM series, GE Osmonics) were considered as a representative range for NF–tight UF range membranes. The intrinsic membrane resistances for the 300 Da, 2000 Da and 8000 Da MWCO membranes were 9.0 × 10^13^ ± 1.1 × 10^13^ m^−1^, 8.1 × 10^13^ ± 1.1 × 10^13^ m^−1^ and 2.2 × 10^13^ ± 2.0 × 10^12^ m^−1^, respectively. The transmembrane pressure applied to the vessels was kept constant at 40.0 ± 1.0 psi (i.e., 2.8 ± 0.1 bar). The tests were conducted at room temperature (23 ± 1 °C). The membrane cells were operated in a pseudo dead-end mode with a continuous bleed that introduced a very low and constant cross-flow of less than 0.006 m/min along the membrane surface. The bleed line allowed the liquid to be purged from the membrane cell when the feed was switched, while still providing pseudo dead-end operation. Based on preliminary tests, this cross-flow velocity had a negligible effect on fouling and concentration polarization (results not presented). All filtration tests were conducted in triplicate in three sequential phases:

1. Pre-clean water filtration phase (for a minimum of 3 h).
2. Filtration phase with model raw waters (for approximately 3 h).
3. Post-clean water filtration phase (for a minimum of 45 min).

The filtration tests were conducted in these three sequential phases to quantify the contribution of CP and fouling to the increase in the resistance to permeate flow, as discussed in more detail in Section 3.2.

Immediately prior to the start of the filtration phase with model raw water, the drain valve was opened and the content of the flow cell was purged, ensuring that the concentration of NOM in the flow cell at the start of the filtration of model raw water, was equivalent to that of the model raw water. The drain was then partially closed to generate a constant low cross-flow velocity of 0.006 m/min.

### 2.2. Data Evaluation and Sample Analyses

The rate of fouling was quantified based on fouling coefficients obtained from standard filtration laws fitted to the experimental data collected during the filtration of model raw water [59]. Based on the minimum R^2^ value for all conditions investigated in the present study, the permeate flux could be best modeled by assuming that fouling was predominantly due to the formation of a cake layer on the membrane surface (see Equation (1); results not presented). Therefore, the results are presented in terms of a cake fouling coefficient (*k_c_*) (see Table 1).

(1) $$J=\frac{J_{0}^{\prime }}{1+k_{c}\;\times \;\frac{\mu }{\Delta P}\;\times \;V\times J_{0}^{\prime }\;}\left[m^{3}{\;m}^{-2}{\;s}^{-1}\right]$$

In Equation (1), *J* represents the permeate flux [m^3^ m^−2^ s^−1^], *V* the specific volume of water filtered [m^3^ m^−2^], µ the dynamic viscosity [Pa s] and ∆*P* the trans-membrane pressure [Pa]. The initial flux $J_{0}^{\prime }$ was defined as the measured clean water flux (*J*_0_), minus any rapid decrease observed in the flux within the first few minutes of a filtration test. Because CP is expected to develop within the first minutes of filtration, the difference between *J*_0_ and $J_{0}^{\prime }$ was assumed to be due to CP [39,45]. The estimated cake fouling coefficient *k_c_* [m^−1^ m^−3^ m^2^] quantifies the increase in resistance [m^−1^] per specific volume filtered [m^3^ m^−2^].

Size exclusion chromatography (SEC) was performed using High Performance Liquid Chromatography (HPLC) (Perkin Elmer, Burnaby, BC, Canada) with DOC detection for the analysis of NOM in the feed and permeate. The method used was adopted from Huber et al. [51]. A TSK HW-50S column (Tosoh, Tokyo, Japan) was used as the stationary phase, and a phosphate buffer (2.5 g/L KH_2_PO_4_ + 1.5 g/L Na_2_HPO_4_·H_2_O) was used as the mobile phase. The sample injection volume and flow rate were 1 mL and 1 mL/min, respectively. A GE Sievers 900 Turbo Portable TOC Analyzer (GE Sievers, Boulder, CO, USA) with a sampling rate of 4 s and a detection range of 0.2 mg C/L to 10 mg C/L was used as the DOC detector.

## 3. Results

### 3.1. NOM Rejection

**Figure 2:**
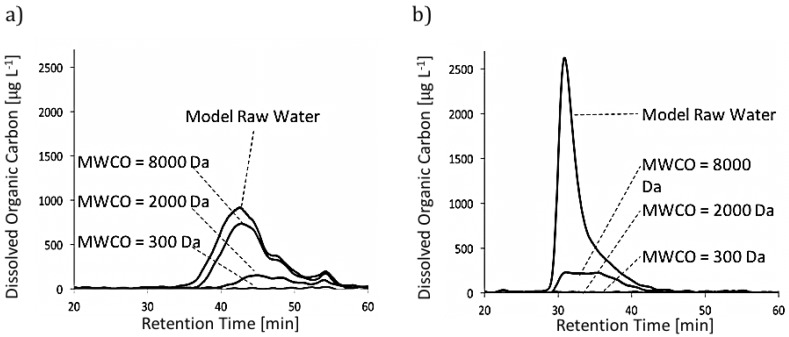
Typical size exclusion chromatograms of model raw water and permeate samples: (**a**) model raw water containing Suwannee River natural organic matter (SRNOM) at 10 mg/L; (**b**) model raw water containing alginate at 10 mg/L. Dalton values correspond to the molecular weight cut offs (MWCOs) of the different membranes; note that the retention time of the chromatograms is inversely proportional to the log of the molecular weight.

The amount of material rejected by the membranes was defined as the difference between material present in the feed (i.e., the model raw water) and material present in the permeate. As illustrated in Figure 2, the membranes with MWCOs of 300 Da and 2000 Da could effectively reject all of the alginate present in model raw waters, and the membrane with a MWCO of 8000 Da could reject most of the alginate present in model raw waters. This was expected because the molecular weight of alginate is greater than 10,000 Da [39,51] and the MWCOs of all the membranes considered are lower than 10,000 Da (i.e., ranging from 300 Da to 8000 Da). Only a small amount of the larger constituents in SRNOM was rejected by the membrane with a MWCO of 8000 Da. The extent of the rejection increased as the MWCO of the membranes decreased, with all of the organic material being rejected by the membrane with a MWCO of 300 Da. Again, this was expected, because most of the constituents of SRNOM have a molecular weight in between 300 Da and 8000 Da [49,50].

Note that NOM in most surface waters consists mainly of humic substances (i.e., lower molecular weight NOM) [32]. Because effective NOM removal is one of the main motivations behind implementing NF and tight UF membranes in drinking water treatment, membranes with lower MWCOs (i.e., <2000 Da) are recommended for this application.

### 3.2. NOM Fouling and Concentration Polarization

**Figure 3:**
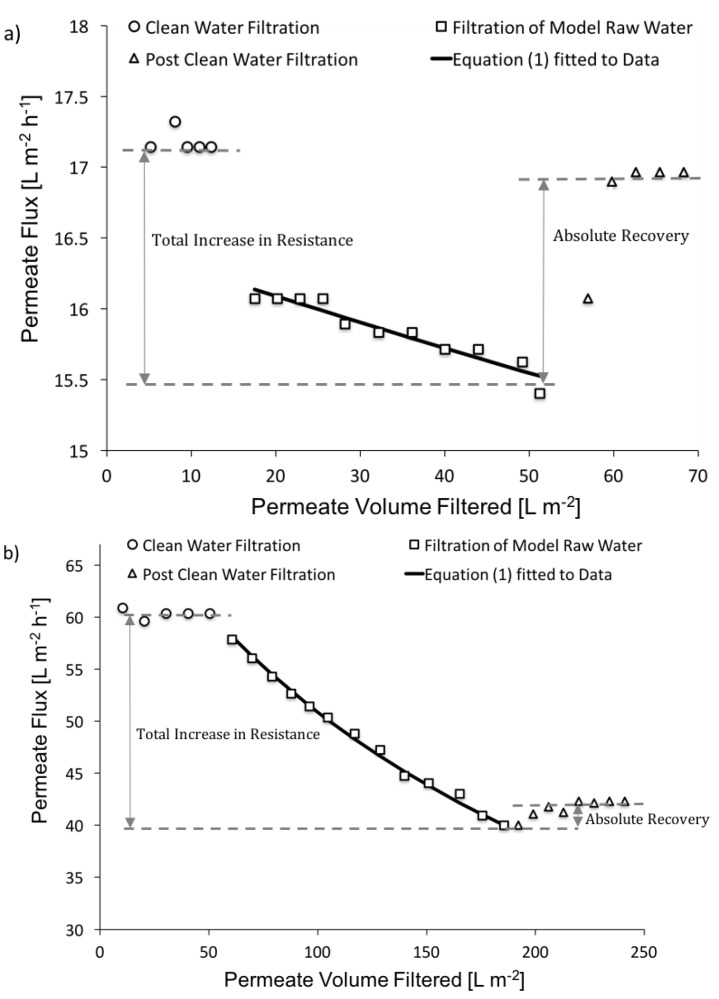
Typical results from filtration tests: (**a**) MWCO = 300 Da and SRNOM at 5 mg/L of DOC, (**b**) MWCO = 8000 Da and alginate at 5 mg/L of DOC.

Typical results from the filtration tests are presented in Figure 3. For all conditions investigated, the permeate flux remained constant during the pre-clean water filtration phase, then decreased over time (i.e., volume filtered) when filtering model raw waters, and increased during the post-clean water filtration phase.

As previously discussed, CP is characterized by an equilibrium between the convective transport of material towards the membrane, and the diffusive transport of retained material away from the membrane. Steady state is expected to be established within minutes once filtration of model raw water begins [39,45]. Hence, the rapid initial decrease in the permeate flux when filtering model raw waters was attributed to CP, and the subsequent slower longer term decrease in permeate flux was attributed to fouling.

Fouling occurs if the permeation drag is greater than the forces acting on potential foulants away from the membrane [22]. Because the permeation drag remains constant when switching from the model raw water filtration phase to the post-clean water filtration phase, no back transport and no recovery in resistance is expected during post-clean water filtration. However, a recovery in resistance due to CP is expected during post-clean water filtration. This is because a concentration gradient of retained material is still present, for a relatively short period of time, after switching from the model raw water filtration phase to the post-clean water filtration phase. During this time, the convective transport of material towards the membrane is zero (i.e., clean water filtration), while the diffusive transport of retained material away from the membrane surface is greater than zero, resulting in a reduction in resistance (i.e. recovery in resistance). Note, that if CP becomes extensive, and a cake/gel layer is formed, the recovery in resistance during post-clean water resistance will be limited [26,46,47,48]. Therefore, although a high recovery during post-clean water filtration suggests that CP likely dominates the increase in resistance due to permeate flow, other indicators of fouling and CP must be considered when interpreting the results.

As previously discussed, for all conditions investigated, the permeate flux could be best modeled assuming fouling was predominantly due to the formation of a cake layer on the membrane surface (see Section 2.2). The fit of the cake fouling model (Equation (1)) to typical data is presented in Figure 3. For all conditions investigated, the extent of fouling was quantified with respect to the fouling coefficient, as discussed in Section 3.2.1, and the contributions of CP and fouling to the increase in filtration resistance was analyzed as discussed in Section 3.2.2.

#### 3.2.1. Contribution of Fouling to the Total Increase in Filtration Resistance

The fouling coefficients (*k_c_*) for the different experimental conditions investigated are summarized in Table 1. When filtering model raw waters containing alginate, the fouling coefficients were similar for the membranes with MWCOs of 300 and 2000 Da and only slightly lower (and not statistically different) for the membrane with the MWCO of 8000 Da. These results are consistent with those presented in Section 3.1, where the rejection of alginate was similar when filtering with membranes with MWCOs of 300 Da and 2000 Da MWCO, and slightly lower when filtering with the membrane with a MWCO of 8000 Da. Considering the substantially different initial permeate fluxes for the membranes with different MWCOs, and the similar fouling coefficients observed for all membranes, it can be concluded that the fouling coefficient was independent of the initial permeate flux. Further, the fouling coefficient was proportional to the concentration of alginate in the model raw water being filtered (i.e., the fouling coefficient doubled when the concentration of alginate in the raw water was twice as high), suggesting that for all membranes, fouling was similar and predominantly due to convective transport and the accumulation of a cake layer with similar characteristics for all membranes. These results are also consistent with the fact that the molecular weight of alginate is greater than the MWCO of all membranes considered (see Section 3.1).

When filtering model raw waters containing SRNOM, the fouling coefficient was substantially higher for the membrane with a MWCO of 300 Da than for the membranes with MWCOs of 2000 and 8000 Da. This is again consistent with the higher rejection of SRNOM by the membrane with a MWCO of 300 Da, than the membranes with MWCOs of 2000 and 8000 Da. It should be noted that the fouling coefficient was similar for the membranes with MWCOs of 2000 and 8000 Da, even though the rejection of organic material present in SRNOM was greater for the membrane with a MWCO of 2000 Da, than for the membrane with a MWCO of 8000 Da. For the membrane with a MWCO of 8000 Da, the fouling coefficient was proportional to the concentration of SRNOM in the model raw water. However, for the membranes with MWCOs of 300 and 2000 Da, the fouling coefficient was not proportional to the concentration of SRNOM in the model raw water. The deviation from proportionality increased as the MWCO of the membrane decreased. These results indicate that, in contrast to filtering model raw water containing alginate, when filtering model raw water containing SRNOM (i.e., using membranes with low MWCOs), fouling was not only governed by the convective transport of material towards the membrane, but also likely by the formation of a CP-induced cake/gel layer at the membrane surface, as discussed in Section 3.2.2.

When filtering model raw waters containing a mixture of SRNOM and alginate, the fouling behavior was similar to that of model raw water containing SRNOM, suggesting that the higher percentage of low molecular weight NOM (i.e., 80% low molecular weight NOM versus 20% high molecular weight NOM) governed the fouling behavior.

#### 3.2.2. Contribution of CP to the Total Increase in Filtration Resistance

**Figure 4:**
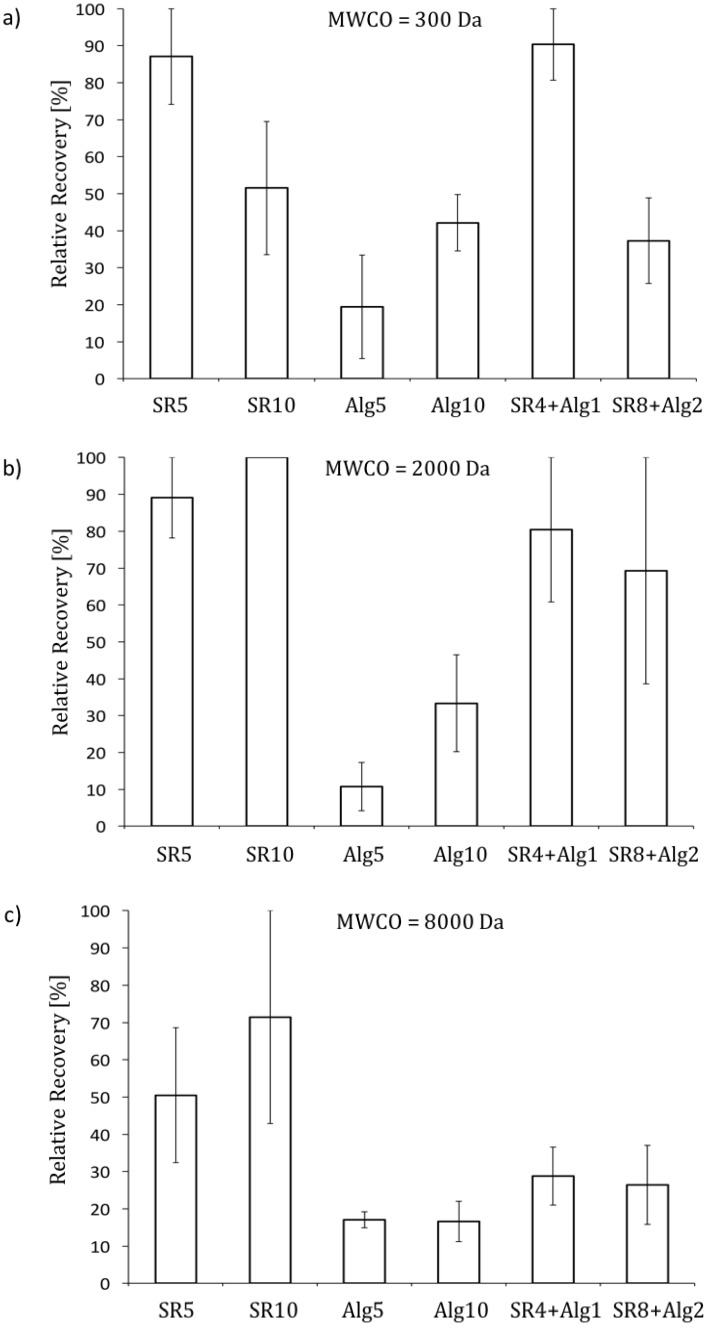
Relative recovery during post-clean water filtration (**a**) MWCO = 300 Da; (**b**) MWCO = 2000 Da; (**c**) MWCO = 8000 Da; SR5: SRNOM at 5 mg/L; SR10: SRNOM at 10 mg/L; Alg5: Alginate at 5 mg/L; Alg10: Alginate at 10 mg/L; SR4 + Alg1: SRNOM at 4 mg/L + alginate at 1 mg/L; SR8 + Alg2: SRNOM at 8 mg/L + alginate at 2 mg/L; error bars correspond to observed minimum/maximum values).

The contribution of CP to the total increase in resistance during filtration of the model raw waters was quantified by the relative recovery. The relative recovery was defined as the ratio of the absolute recovery to the total increase in resistance during filtration of model raw water (Figure 3). The total increase in resistance was calculated based on the difference between the permeate flux during a pre-clean water filtration phase, and at the end of a filtration of model raw water phase. The absolute recovery was calculated as the difference between the permeate flux at the end of a filtration of model raw water phase, and at the end of a post-clean water filtration phase. Figure 4 illustrates the relative recoveries for the different conditions investigated.

The relative recovery generally increased as the MWCO of the membrane decreased. Also, the relative recovery generally increased when the concentration of NOM in the raw water increased. When filtering model raw water containing alginate, the relative recovery was generally low, ranging from approximately 10% to 40%. Also, the fouling coefficient increased proportionally with the concentration of alginate in the model raw water (see Section 3.2.1). This, combined with the low relative recoveries, suggest that the increase in resistance to permeate flow was dominated by fouling when filtering model raw waters containing alginate, at all of the considered concentrations (i.e., 5 mg L^−1^ and 10 mg L^−1^). However, when filtering model raw waters containing SRNOM, the relative recovery was generally higher, ranging from approximately 50% to 100%. The greater relative recoveries when filtering model raw waters containing SRNOM, rather than alginate, was attributed to the differences in the diffusion coefficients of these types of NOM. Diffusion coefficients of 2.2 × 10^−10^ to 3.8 × 10^−10^ m^2^ s^−1^ have been reported for humic substances [29], which account for most of the organic material in SRNOM, while that for alginate is in the order of 9.4 × 10^−11^ m^2^ s^−1^, an estimate based on Dextran T-70 [29]. The diffusive transport of retained material from the membrane back into solution is expected to increase relative to the molecular diffusion [29]. Also, the permeation drag is expected to be greater for alginate than for SRNOM because of the larger size of alginate compared to SRNOM [22]. The relative recovery for the membrane with a MWCO of 300 Da was lower when the concentration of SRNOM in the model raw water increased (i.e., from 5 to 10 mg/L). Under these conditions, the concentration at the membrane surface is expected to be high, and likely resulted in the formation of a cake/gel layer at the membrane surface [26,46,47,48]. Once formed, the resistance offered by the cake/gel layer could not be reversed during post-clean water filtration, which is consistent with previous research where humic substances fouling was observed to be irreversible [25,26,29,35].

When filtering model raw waters containing a mixture of SRNOM and alginate, the relative recovery was similar to that of model raw waters containing SRNOM, again suggesting that the higher percentage of low molecular weight NOM (i.e., 80% low molecular weight NOM versus 20% high molecular weight NOM) was mainly responsible for the observed changes in permeate flux.

**Figure 5:**
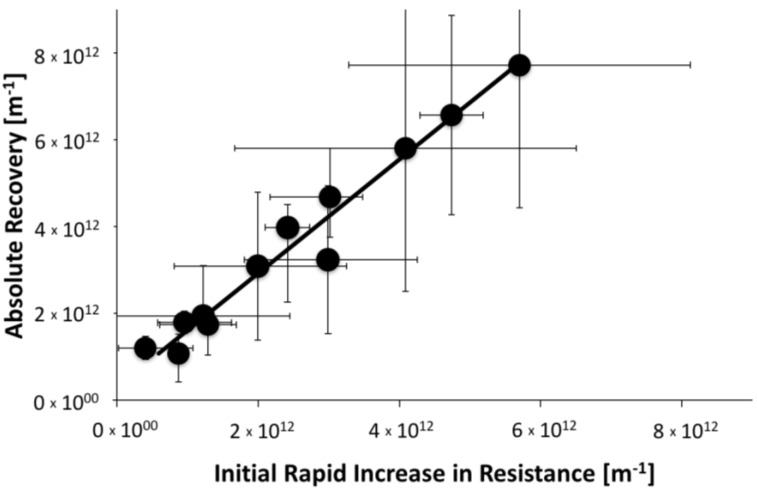
Correlation between Absolute Recovery and Initial Rapid Increase in Resistance (model raw waters containing SRNOM and a mixture of SRNOM + alginate; solid line: linear regression, R^2^ = 0.97, slope = 1.31 ± 0.35, intercept = 3 × 10 ^11^; *p* = 0.05; error bars correspond to minimum/maximum values).

As illustrated in Figure 5, when filtering model raw waters containing SRNOM or a mixture of SRNOM and alginate, a linear correlation was observed between the initial rapid increase in resistance during model raw water filtration and the absolute recovery during post-clean water filtration (R^2^ = 0.97). When filtering model raw waters containing alginate, no correlation was observed (R^2^ = 0.04, results not shown). The correlation supports the assumption that the initial rapid increase in resistance is due to CP rather than fouling, that CP develops rapidly within the first minutes of filtration when filtering raw waters containing NOM, and confirms that the relative recovery observed when filtering model raw waters containing SRNOM can be attributed to CP. In addition, these results confirm that the total increase in filtration resistance is mainly impacted by CP when filtering model raw waters containing SRNOM.

#### 3.2.3. Overall Impact of Fouling and CP to the Increase in Resistance to Permeate Flow

Overall, the results suggest that when filtering model raw waters containing alginate, the total increases in the filtration resistance is dominated by fouling, while the total increase in filtration resistance is dominated by CP when filtering model raw waters containing SRNOM (i.e., humic substances, lower molecular weight NOM). The impact of CP is greatest for membranes with lower MWCOs (i.e., <2000 Da), which are recommended for the effective removal of NOM in drinking water treatment applications. Filtration using membranes with higher MWCOs (e.g., 8000 Da) is not prone to CP because the lower molecular weight NOM, responsible for CP, is not effectively retained.

It should be noted that when the impact of CP is extensive (i.e., filtration of raw waters containing mainly humic substances/low molecular weight NOM with low MWCO membranes), the increase in resistance is much greater than when filtration was dominated by fouling. For this reason, and because NOM in surface waters consist predominantly of humic substances [32], operation with control measures to continuously limit CP, such as cross-flow operation, should be considered for drinking water treatment using NF and tight UF range membranes. Dead-end operation, which is typically used for drinking water production by UF, is not recommended because it cannot continuously limit CP.

Future research will investigate the impact of control measures (e.g., cross-flow) and different membrane configurations (e.g., hollow fiber versus spiral wound membrane configurations) to mitigate CP and fouling in NF and tight UF membrane systems applied to the treatment of drinking water.

## 4. Conclusions

The following four main conclusions can be made, based on the results of the present study:

1. Since NOM in most surface waters consists mainly of humic substances (i.e., lower molecular weight NOM), and because effective NOM removal is likely to be one of the main motivations for implementing NF and tight UF range membranes in drinking water treatment, membranes with lower MWCOs (i.e., <2000 Da) are recommended for this application.
2. When filtering raw waters containing high concentrations of alginate (i.e., polysaccharides, high molecular weight NOM), the total increase in the filtration resistance is mainly dominated by fouling; when filtering raw waters containing predominantly humic substances (i.e., lower molecular weight NOM), the total increase in filtration resistance is mainly dominated by CP. The impact of CP is greatest for membranes with low MWCOs (i.e., <2000 Da).
3. When filtering raw waters containing high concentrations of humic substances (i.e., 10 mg L^−1^) with the membrane with the lowest MWCO (i.e., 300 Da), the impact of CP became extensive and the increase in resistance was greater than that observed for any other experimental condition considered, likely due to the formation of a CP-induced cake/gel layer.
4. Operations with control measures to continuously limit CP, such as cross-flow operation, should be considered in drinking water applications using NF and tight UF range membranes. Dead-end operation, which is typically used for drinking water production using UF, is not recommended because it cannot continuously limit CP.

## Figures and Tables

**Table 1:** Fouling coefficient *k_c_* for different filtration tests (ranges correspond to observed minimum/maximum values).

NOM Constituents	NOM Concentration [mg/L]	Fouling Coefficient *k_c_* [10^11^ m^−1^ m^−3^ m^2^] Average (Range)		
		MWCO = 300 Da	MWCO = 2000 Da	MWCO = 8000 Da
SRNOM	5	0.7 (0.5–0.9)	0.1 (0–0.1)	0.1 (0.1–0.1)
	10	3.5 (3.5–3.5)	0.6 (0–1.1)	0.2 (0.2–0.2)
Alginate	5	1.1 (0.8–1.6)	0.8 (0.5–1.1)	0.8 (0.7–0.9)
	10	2.1 (2.0–2.2)	2.1 (1.6–2.4)	1.6 (1.5–1.7)
SRNOM + Alginate	4 + 1	0.7 (0.4–1.0)	0.2 (0.1–0.3)	0.2 (0.2–0.2)
	8 + 2	5.1 (4.7–5.5)	1.2 (0.7–2.0)	0.5 (0.5–0.5)
